# Variococele of Twelve Years’ Duration; Great Pain in the Testicle; Operation by Wire Ligature; Cure

**Published:** 1864-01

**Authors:** 


### VARICOCELE OF TWELVE YEARS' DURATION; GREAT PAIN IN THE TESTICLE; OPERATION BY WIRE LIGATURE; CURE.—

H. G., æt. 24, a butcher, was admitted into Westminster Hospital, under the care of Mr. B. Holt, April 16, 1863, suffering from varicocele of twelve years' duration. He states that for many years he has been accustomed to ride for several hours a day, and frequently without a saddle; that he does not remember to have met with any accident, but that his business required him to stand for a considerable period of the day; that he always felt weak, but has not been laid up with any serious illness. Twelve years since he complained of pain in his left testicle, which was increased upon exertion, and occasionally was very severe at night. He consulted a surgeon who advised him to wear a suspensory bandage; but although he had continued to do so, the pain in the testicle had latterly so much increased as to compel him to abandon his work. Upon examination, the usual tortuous and congested condition of the veins was detected behind the testicle, which, however, could be emptied by placing him in a horizontal position; but when pressure was applied over the external abdominal ring, and the patient was desired to resume the perpendicular, the veins were more swollen than before. The vas deferens could be easily isolated.

Mr. Holt determined to tie the veins with the wire suture by the subcutaneous method; and the patient being placed under the influence of chloroform, and the vas deferens isolated, a needle armed with the wire ligature was passed through the scrotum behind the diseased veins, and made to perforate the scrotum on the inner side. The needle was now made to enter at the same opening, and the wire was passed in front of the veins, and so out at the first puncture; thus no skin was included in the wire loop, but merely the veins and some areolar tissue. The wire was now twisted, and the same proceeding was adopted a little lower down, by which the veins were thoroughly included in two ligatures. The wires were now shortened, the intention being to subject the veins to compression for three or four days, and then to remove them by simply untwisting them. For the next three days the patient was comfortable, and did not complain of pain. There was slight swelling, and tenderness when the part was touched, but no other pain. On the fourth day Mr. Holt intended untwisting the wires as before described; but the twisted portion was so deeply imbedded that he determined to cut both ends short, and leave them as permanent compressors. The punctures healed in two days, and the wires remained, not exciting any irritation or disturbance, and the patient was able to work without inconvenience. He was detained in the hospital for a fortnight that Mr. Holt might see whether any inconvenience was experienced; but none having occurred at the expiration of that time, he was discharged, but requested to present himself from time to time that the result might be watched. The veins between the ligatures were entirely obliterated, and the patient described himself as perfectly free from any pain in the testicle. He has presented himself occasionally as requested. The wires can be felt, but they do not occasion any inconvenience, and he has resumed his work with a perfect immunity from pain.

Mr. Holt remarked that this was a case from which probably some instruction might be derived. He stated it was not his intention, in the first instance, to have left the wires around the veins; but as there was some little difficulty in their removal, he determined to retain them, as in all probability they would not excite irritation, and they would positively prevent any recurrence of the disease. He considered it was a practice that might be repeated with advantage. The pain of the operation was merely that which resulted from the two punctures in the skin, and the patient had not otherwise suffered, as was almost always the case when any portion of the integument was subjected to pressure. The operation was in itself of the easiest possible kind to perform, and he did not consider the patient need be confined to bed. Should future cases be attended with a like favorable issue, the proceeding would become general, and the pain and time of the patients be considerably abridged, while the question of recurrence would be entirely set at rest, such an occurrence being impossible so long as the wires acted as compressers.—*Lancet*, *Aug.* 16, 1863.

